# Nonpoint source pollution measures in the Clean Water Act have no detectable impact on decadal trends in nutrient concentrations in U.S. inland waters

**DOI:** 10.1007/s13280-023-01869-6

**Published:** 2023-06-23

**Authors:** Nathan Tomczyk, Laura Naslund, Carolyn Cummins, Emily V. Bell, Phillip Bumpers, Amy D. Rosemond

**Affiliations:** 1 Odum School of Ecology, University of Georgia, 140 E. Green St., Athens, GA 30602 USA; 2 School of Public & International Affairs, University of Georgia, 415 Baldwin Hall, Athens, GA 30602 USA

**Keywords:** Ammonium, Nitrate, Phosphorus, Policy evaluation, Section 303, Section 319

## Abstract

The Clean Water Act (CWA) of 1972 regulates water quality in U.S. inland waters under a system of cooperative federalism in which states are delegated implementation and enforcement authority of CWA provisions by the U.S. Environmental Protection Agency. We leveraged heterogeneity in state implementation of the CWA to evaluate the efficacy of its nonpoint source provisions in reducing nutrient pollution, the leading cause of water quality impairment in U.S. inland waters. We used national survey data to estimate changes in nutrient concentrations over a decade and evaluated the effect of state-level policy implementation. We found no evidence to support an effect of (i) grant spending on nonpoint source pollution remediation, (ii) nutrient criteria development, or (iii) water quality monitoring intensity on 10-year trends in nutrient concentrations. These results suggest that the current federal policy paradigm for improving water quality is not creating desired outcomes.

**Supplementary Information:**

The online version contains supplementary material available at 10.1007/s13280-023-01869-6.

## Introduction

At a time when rivers caught fire, the United States (U.S.) Congress passed legislation with the monumental goals of attaining water quality protective of recreation and wildlife within 11 years and eliminating all pollutant discharges into navigable waters within 13 years (33 U.S.C.§1251). Today, the Clean Water Act of 1972 (CWA) remains the primary legislation regulating freshwater quality in the U.S. The CWA operates on a principle of cooperative federalism, whereby the U.S. Environmental Protection Agency (EPA) delegates much of the implementation and enforcement authority of the law to the states (Malloy 2011). A purported advantage of cooperative federalism is that states can act as “laboratories of democracy,” developing and testing innovations in law and policy that can inform nationwide practice. The heterogeneity in federal policy implementation across states also provides a useful opportunity for evaluating the efficacy of particular statutes, and has underpinned previous analyses of both the CWA and the Clean Air Act (Keiser and Shapiro 2018; Aldy et al. 2022). However, the efficacy of CWA provisions in reducing excess nitrogen (N) and phosphorus (P) pollution has not, to our knowledge, been evaluated systematically, despite nutrient pollution being the leading cause of water quality impairment in the U.S. (U.S. EPA 2019, 2022c). We address this gap by quantifying state-specific trends in nutrient concentrations in streams, rivers, and lakes over 10 years and evaluating the association of these trends with state-level implementation of CWA provisions.

Nitrogen (N) and phosphorus (P) exceed concentrations that are protective of aquatic life in most US streams (Manning et al. 2020) and can cause harmful algal blooms (Conley et al. 2009; Paerl et al. 2011), impair drinking water (Temkin et al. 2019), and alter the structure and function of aquatic ecosystems (Elser et al. 2007; Rosemond et al. 2015). In the U.S., 58% of stream and river miles are in poor condition due to excess P and 43% due to excess N (U.S. EPA 2019). A similar proportion of U.S. lakes are impaired for P (40%) and N (35%) (U.S. EPA 2022c). Nutrients enter freshwater ecosystems through diverse sources and pathways including agricultural sources from cultivated land, animal feeding and grazing, atmospheric deposition from fossil fuel combustion, and sources associated with human infrastructure, such as waste treatment and stormwater runoff (Brown and Froemke 2012). The CWA provides different mechanisms for regulating pollution from point sources, those originating from a “discrete conveyance” like wastewater treatment plant discharges, and “nonpoint sources” like agricultural runoff, which are extensive and spatially diffuse (33 U.S.C.§1362; Salzman and Thompson 2019). Because nonpoint sources dominate N and P inputs to surface waters in the U.S. (Sabo et al. 2021), our analysis focused on the efficacy of nonpoint source pollution provisions in the CWA for reducing nutrient pollution by leveraging the heterogeneity in the implementation of these provisions at the state level.

Sections 303 and 319 are the primary provisions of the CWA which aim to reduce nonpoint source pollution. Section 303 requires states to designate uses for each waterbody within its borders. At a minimum, designated uses must be protective of wildlife and recreation in and on the water, but a state can downgrade a waterbody to a lower standard with the permission of the EPA. States must then identify water quality standards to support these designated uses. These standards may be numeric or narrative; however, in 1998, the EPA promulgated a national plan to develop quantitative state water quality standards for nutrients (U.S. EPA 1998). More than 20 years later, the adoption of numeric nutrient criteria by the states is heterogeneous (U.S. EPA 2022e). Establishing numeric nutrient criteria has long been recognized by the EPA as an important step for identifying and addressing nutrient pollution (U.S. EPA 2009). For this reason, we explored the impact of numeric nutrient criteria establishment on state water quality trends.

Under Sect. 303 of the CWA, states must also identify waterbodies which do not attain their designated uses after adjusting permits for point source discharges (33 U.S.C.§1313). For each of these “quality-limited” waterbodies, the state must determine the total maximum daily load (TMDL) of each pollutant that is allowable to attain the waterbody’s designated use. However, states are not compelled to implement identified TMDLs for quality-limited waterbodies (Salzman and Thompson 2019). Nonetheless, monitoring to identify quality-limited waterbodies *is* required by the CWA and is the first step in remediating polluted waters. Thus, we explored the association between the intensity of monitoring for the TMDL program and state water quality trends.

The 1987 CWA amendment established Sect. 319, which designates federal grant money to support state-level plan implementation for a suite of activities, including but not limited to monitoring of nonpoint source implementation projects, technical assistance, technological transfer, and education (U.S. EPA 2022a). Current EPA guidelines require states to have up-to-date nonpoint source management programs to receive 319 grant funds. EPA guidance specifies that these programs must articulate goals and strategies to address nonpoint source pollution (U.S. EPA 2013). The EPA regional offices must also determine that a state has made “satisfactory progress” in achieving the goals of their management program in order to be eligible for 319 funds (U.S. GAO 2012). Activities funded by the 319 program can impact nutrient concentrations directly through new nonpoint source remediation projects or indirectly through, e.g., community education initiatives. Thus, we explored the relationship between 319 expenditures and state water quality trends.

Identifying effective policies for reducing nutrient pollution is a critical step to achieve the goals set forth in the CWA. The heterogeneity in CWA policy implementation of numeric nutrient criteria, monitoring intensity, and grant spending across states creates an opportunity to connect policy implementation to nutrient pollution outcomes in US waters. Because the cooperative structure of the CWA delegates implementation and enforcement authority to the states, the state is a critical unit of study to examine relationships between policy implementation and outcomes. To quantify state-level trends in mean nutrient concentrations over the past decade (~ 2007 to  ~ 2019), we used nutrient concentration data from lakes/ reservoirs and streams/ rivers collated in the National Aquatic Resource Surveys. We hypothesized (H1) that states with more complete (i.e., more water bodies covered) numeric nutrient criteria for longer durations experienced greater reductions in nutrient concentrations over a decade than states with incomplete or nonexistent numeric nutrient criteria. Further, we hypothesized (H2) that states that surveyed more sites through the TMDL program or (H3) had greater total expenditures through the 319 program (relative to the size of the state) experienced greater reductions in nutrient concentrations over the past decade.

## Materials and methods

### Water quality data

To estimate temporal trends in statewide nutrient pollution, we used data from the National Rivers and Streams Assessment (NRSA), and the National Lakes Assessment (NLA). In these assessments, sites within each of the 48 conterminous states were selected probabilistically, facilitating the estimation of state-level means for various parameters (U.S. EPA 2019, 2022c). We focused on dissolved nitrate (NO_3_^−^-N), dissolved ammonium (NH_4_^+^-N), total nitrogen (TN), and total phosphorus (TP) in streams and rivers and on TN and TP in lakes and reservoirs. The stream data were collected in 2008–2009, 2013–2014, and 2018–2019 while the lake data were collected in 2007, 2012, and 2017. We excluded all observations with quality control flags (e.g., exceeded storage and shipping times) and only used sampling sites selected probabilistically in our analysis. Samples flagged as below detection limit were set equal to the method detection limit for subsequent analyses.

### Accounting for differences in nutrient inputs

We accounted for changes in nutrient inputs, which may drive changes in mean nutrient concentrations across states, by examining both the status of variables associated with nutrient input near the beginning of the time period of interest (2007) and how they changed during this period (~ 2007 to ~ 2019). First, because the discharge of human waste is an important source of nutrients to aquatic systems (Iverson et al. 2018) and total waste discharge is linked to human population size (Capps et al. 2020), we included two variables: the population in 2010 and the change in human population between 2010 and 2019 in each state (US Census Bureau 2019). Second, we included variables representing primary agricultural drivers of eutrophication: animal feeding operations, fertilizer use, and land use (Glibert 2020). We calculated the total monetary value of fertilizer, lime, and soil conditioners purchased in 2007 and 2017, the total monetary value of animal feed purchased in 2007 and 2017, as well as the amount of agricultural land use in 2008 and 2019 (Dewitz 2019), in each state as a proxy for agricultural nutrient inputs. We did not adjust these monetary values for inflation as our primary goal was to account for relative differences in nutrient input among states to better estimate the effect of policies and not to make inferences about the effect of nutrient input variables. For each agricultural variable, we included the status of the agricultural variable in 2007 or 2008, and, separately, the change in the variable over the study period (USDA 2017). Finally, we included land cover in each state in 2008 and the change between 2008 and 2019 (Dewitz 2019). We combined low, medium, and high intensity urban land uses into an “urban” land cover category and combined all forested land cover with “barren,” “scrub-shrub,” “herbaceous,” and “wetland” into a land cover category we call “undeveloped.” We normalized each of these variables to the land area in each state that is under state jurisdiction for environmental quality, which is the total area within a state boundary minus the area under federal or tribal jurisdiction. The spatial resolution of the water quality data did not allow us to evaluate policy in sovereign tribal nations, although management in these areas may differ from states and could offer valuable insights into water quality management.

### Policy variables

We predicted that (H1) states which implemented numeric nutrient criteria would experience greater reductions in nutrient concentrations than states without numeric nutrient criteria. We used data recorded by the EPA, which reports whether states had nutrient criteria for N and P for lakes/ reservoirs and streams, and whether these criteria applied to all waterbodies or only some (i.e., statewide/ partial criteria). These data are reported in 2008 and in each year from 2013 to 2020 (U.S. EPA 2022e). For instances where apparent policy changes occurred between 2008 and 2013, we examined the supporting information on the U.S. EPA website to determine the year a policy change occurred. We calculated a nutrient criteria index by aggregating these data across the study interval, giving a state two points for a year in which they had statewide criteria, a single point for a year in which they had partial nutrient criteria, and no points when they had no nutrient criteria. For each nutrient and waterbody type, we used the sum of the nutrient criteria scores across years to represent the status of nutrient criteria during the study period. We recognize that establishing criteria is only a step towards nutrient reduction, but included this in our analysis since it deals directly with oversight of nutrient pollution.

A critical step in reducing nutrient concentrations at the state level is identifying the waterbodies most affected by nutrient pollution. To quantify the effort that states made in identifying impaired waters, we used reporting data from the 303d TMDL program (U.S. EPA 2014). We hypothesized (H2) that states that surveyed a greater number of sites relative to the size of the state experienced greater reductions in mean nutrient concentrations. States are obligated to submit reports to the EPA on a two-year cycle and the U.S. EPA reports one cycle of data for each state; however, the year of the last digitized report varies somewhat from state to state. We used the most recent reports and rescaled the number of sites visited to the area under the jurisdiction of that state (= total area within state boundary—area under federal or tribal jurisdiction).

Lastly, most nutrient pollution originates from nonpoint sources, and we hypothesized (H3) that grant and matching funds spent as part of the CWA 319 program would lead to reductions in nutrient concentrations. We used data from the 319 grants tracking system and summed the total dollar amount of 319 grants from the federal government with state matching funds on projects completed during the study interval (U.S. EPA 2022a). We rescaled this 319 spending to the jurisdictional area of the state.

### Analysis

Our primary interest was exploring how federal policy implemented by states leads to *changes* in mean nutrient concentrations. To this end, we used the trend in mean nutrient concentrations within each state (units of µg L^−1^ year^−1^) as our outcome variable rather than mean nutrient concentration at the end of our study period. We evaluated changes in median concentrations in supplement S2. We estimated rates of change in nutrient concentrations using the *trend_analysis* function in the *spsurvey* package in R (R Core Team 2018; Dumelle et al. 2022). This function allowed us to weight the measured concentration of nutrients using the survey design weights for sites assigned by the survey designers. For instance, sites were sampled evenly within different size classes, but because small waterbodies are more abundant, the stratified sampling design leads to underrepresentation of small waterbodies in the dataset. Thus, smaller, more abundant streams are assigned higher design weights (U.S. EPA 2016; Dumelle et al. 2022). Because there is uncertainty in the trend estimates that should be propagated through our subsequent analysis, we extracted the mean and standard error of the trend estimates for each state and used these values to generate 10,000 normally distributed trend estimates for each state which we fit our subsequent models to individually.

The primary goal of our analysis was to estimate the effects of policy implementation on trends in nutrient concentrations. To accomplish this goal, we needed to account for other factors that may affect trends in nutrient concentrations. We accounted for nutrient inputs by first fitting a global, linear model of input variables as predictors (i.e., population size, population Δ, urban land use, urban land use Δ, undeveloped land use, undeveloped land use Δ, fertilizer use, fertilizer use Δ, animal feed, animal feed Δ) to the mean nutrient trends using ordinary least squares regression. Thus, the general structure of our global model was: nutrient trend ~ nutrient input variables. We transformed each input variable into a z-score to put all variables on a comparable scale (Burnham and Anderson 2002). Then, we conducted model selection by the Akaike Information Criteria (AICc) score for small sample sizes on all models that were a subset of the global model using the *dredge* function in the *MuMIn* package (Bartoń 2020). As our goal was to account for factors driving nutrient inputs and not to create the optimal model to infer the effects of specific input variables on nutrient trends, we selected the model which had the lowest AICc, although in some cases there were models with similar AICc scores. We additionally calculated correlations among policy and nutrient input variables to determine whether correlations among these variables may have confounded our interpretation of the effects of policy. Pearson’s correlations among variables were relatively weak (Table S1), but we completed a complementary analysis in which we determined that including nutrient input variables did not affect the interpretation of policy effects. We present this analysis in supplement S1.

**Fig. 1:**
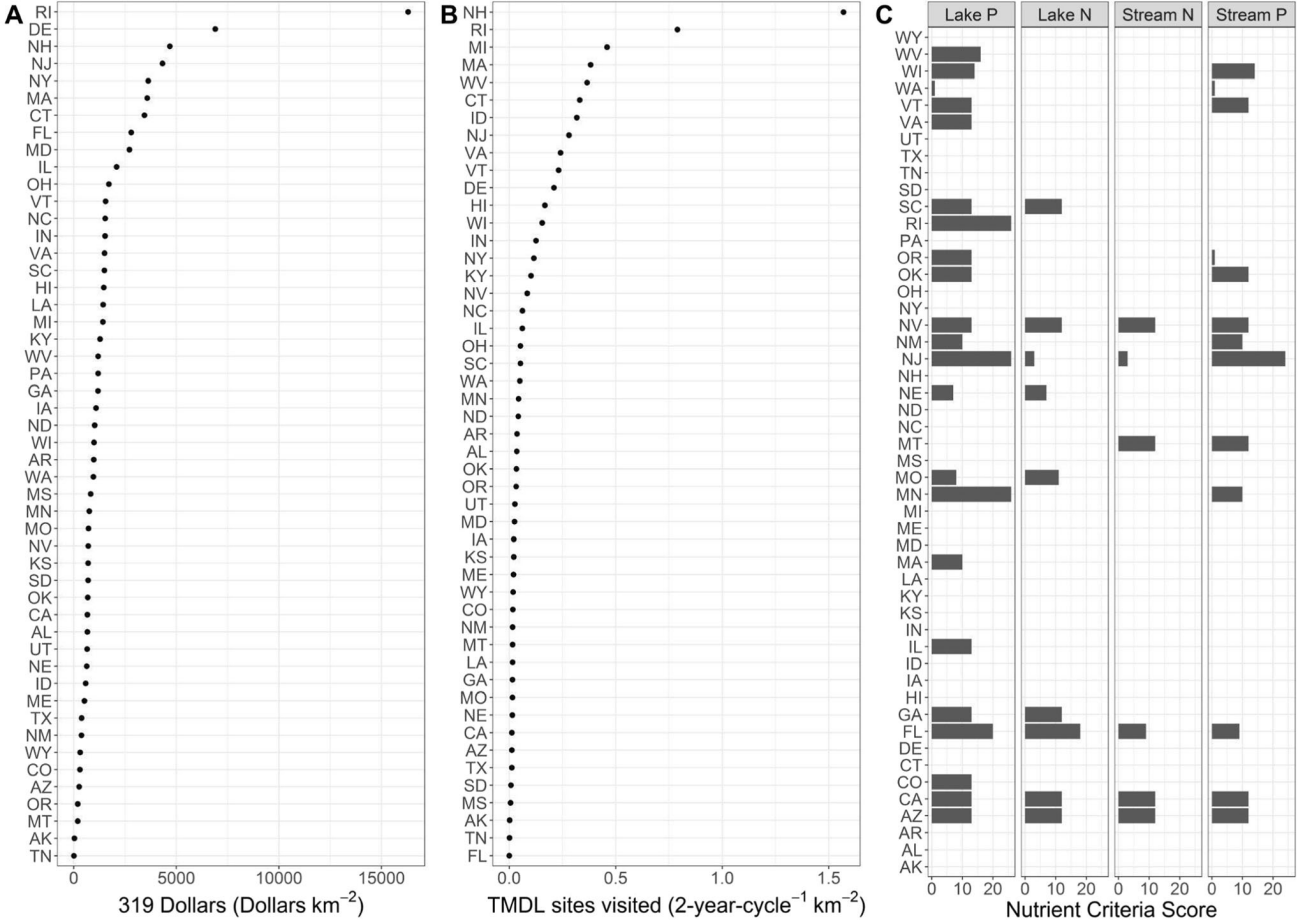
Distribution of total spending under the 319 program (**a**), number of sites visited as part of the TMDL program (**b**), and status of nutrient criteria development (**c**) across the states. Both 319 program spending and TMDL site visits are normalized to the area of state jurisdiction and are presented here on a km^−2^ basis. Nutrient criteria scores are an index and reflect the completeness of guidance (partial vs. statewide) and the duration of time they have been in place. Although Alaska (AK) and Hawaii (HI) are included in this summary figure, they were not included in the policy models due to incomplete coverage by the National Aquatic Resource Surveys during our study period

Because we wanted to estimate the effect of each policy variable, even if the variable did not improve model fit based on AICc, we fit models which included each policy variable separately after selecting the input variables. After determining the most suitable statistical model to represent nutrient inputs, we then added each policy variable separately to this model for each nutrient response-waterbody type pair. We elected to fit the policy variables one at a time because there was some collinearity among policy variables, and our power to detect relationships among variables was limited by the number of states in the continental US. We fit these models to each of the 10,000 sets of nutrient concentration trends over time. From the 10,000 sets of model parameters, we used the mean and standard error of the parameter estimates from each model run to simulate one estimate of the model parameter from the underlying distribution. We summarized the mean and 95% confidence intervals of these estimates to report the effect sizes of policy and input variables on the rate of change in mean nutrient concentrations (µg L^−1^ year^−1^), with negative effects indicating favorable policy outcomes. Additionally, we used model selection to evaluate whether the policy variables improved the performance of the nutrient trend models beyond the input variables alone (our null model). For each of the 10,000 sets of nutrient trend data, we calculated the difference in AICc between the null and policy variable models and averaged these differences across the 10,000 sets. We fit models with z-scored policy variables to aid in the comparison among policy variables, and we fit models with untransformed policy variables to make meaningful estimates of effect sizes (Fig. 1).

## Results

**Fig. 2:**
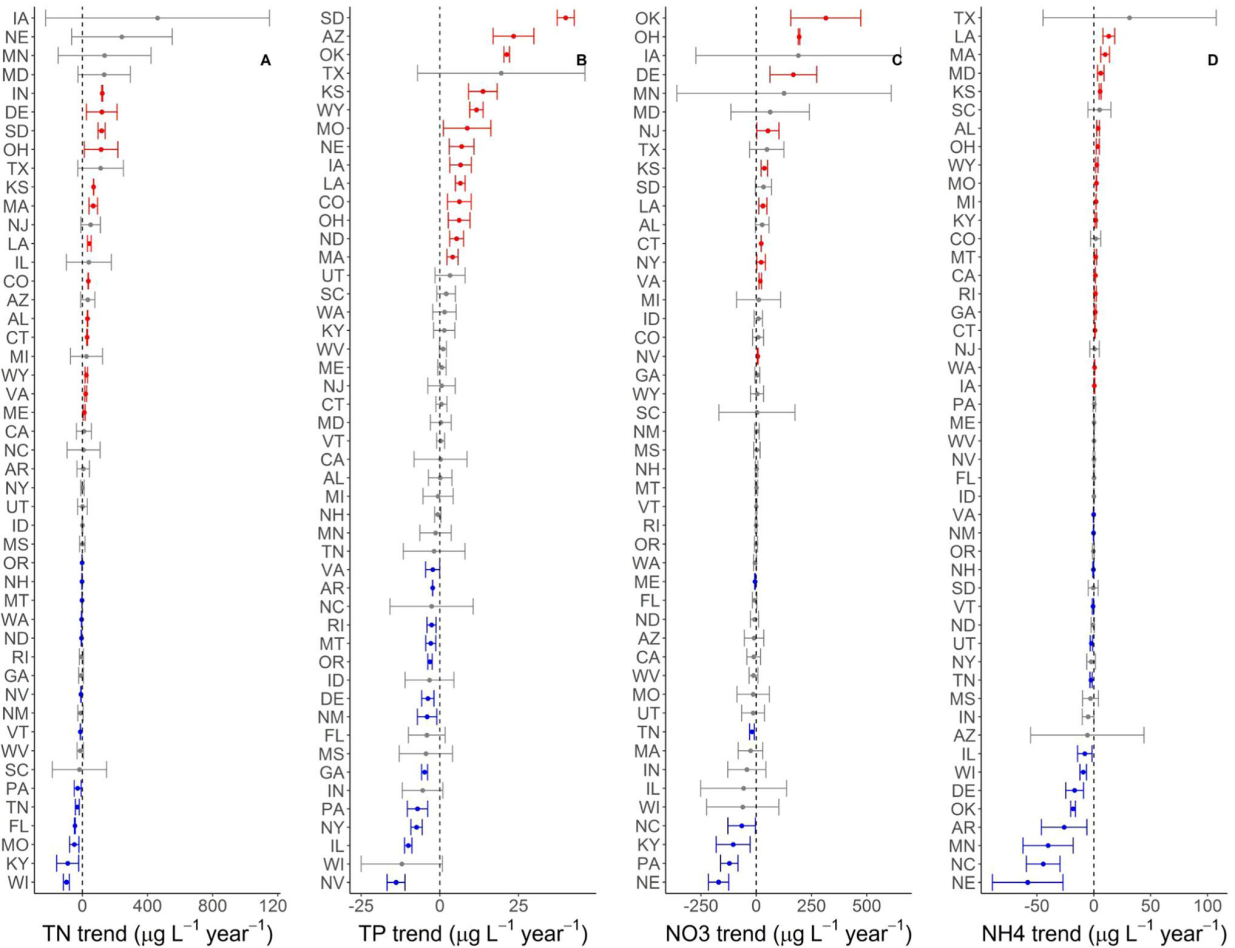
Rate of change in concentration over time (trend) of total nitrogen (TN, **A**), total phosphorus (TP, **B**), nitrate (NO_3_^−^, **C**), and ammonium (NH_4_^+^, **D**) in streams across states which were sampled 2008–2009, 2013–2014, and 2018–2019. In each panel, the states are sorted based on their mean nutrient trend so order of states on the y-axis varies among panels. Points are presented with standard errors and colored based on whether the standard errors overlap zero. Blue points represent significantly declining mean nutrient concentrations, red points represent significantly increasing nutrient concentrations and gray points do not have detectable changes in mean nutrient concentrations

**Fig. 3:**
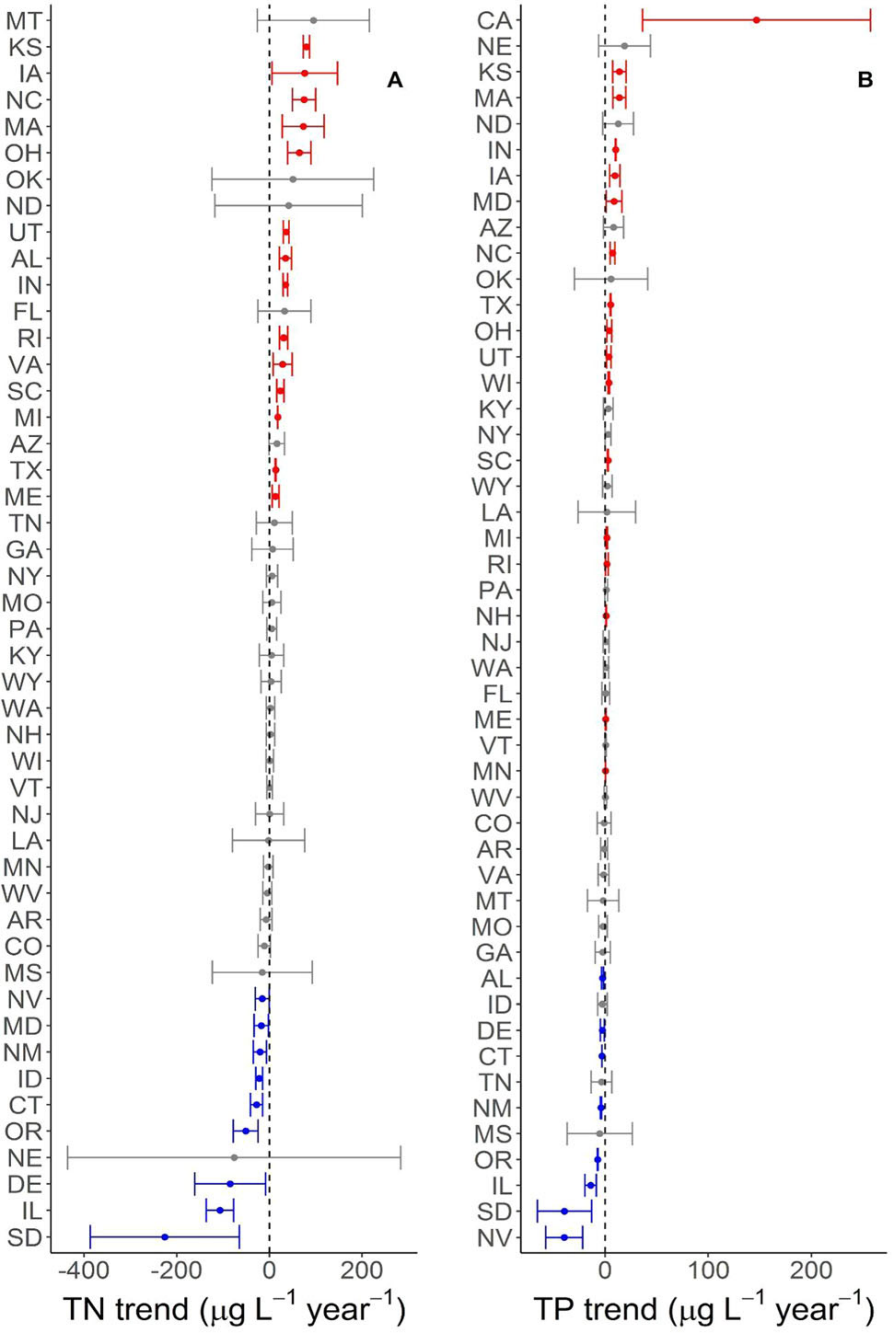
Trends in concentrations of total nitrogen (TN, **A**) and total phosphorus (TP, **B**) in lakes and reservoirs which were sampled 2007, 2012 and 2017. In each panel, the states are sorted based on their mean nutrient trend so order of states on the y-axis varies among panels. Points are presented with standard errors and colored based on whether they overlap zero. Blue points represent significantly declining mean nutrient concentrations, red points represent significantly increasing nutrient concentrations gray points do not have detectable changes in mean nutrient concentrations

Mean nutrient concentrations increased over time in 29% of state-nutrient-waterbody combinations, decreased in 22%, and remained the same in 49% (Figs. 2, 3). There were not strong correlations among trends in nutrient concentrations within states, suggesting that states which experienced large reductions in a given nutrient in either lakes or streams have not necessarily experienced an equally large reduction in the other nutrients or in the same nutrient in the other waterbody type (Table 1). Trends in lake TP and TN along with trends in stream nitrate and stream TN were exceptions, as both pairs were positively correlated (Table 1). Similar to trends in mean concentrations, median concentrations also showed increasing, decreasing, or no-changes in various states (Figures S1, S2).

**Table 1:** Pearson’s correlations among trends in nutrient concentrations for the different nutrients across states. Trends in ammonium, nitrate, total phosphorus (TP), and total nitrogen (TN) among states and in streams and lakes are represented

	Stream ammonium	Stream TP	Stream TN	Lake TN	Lake TP
Stream nitrate	0.24	0.16	0.48	0.1	− 0.09
Stream ammonium		0.17	− 0.21	0.17	− 0.13
Stream TP			0.32	− 0.25	− 0.02
Stream TN				− 0.05	0.2
Lake TN					0.6

**Fig. 4:**
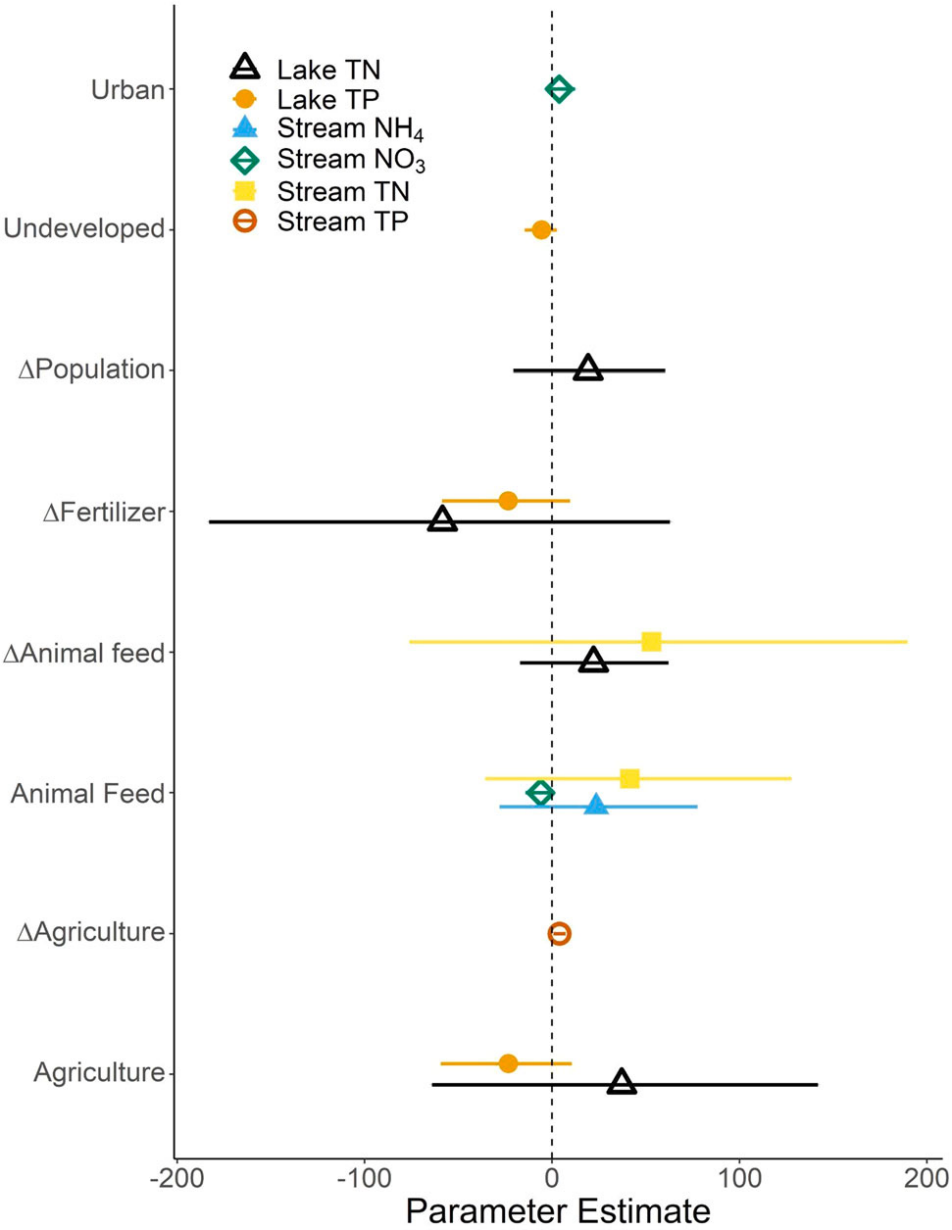
Parameter estimates of the input variables that best described the trends in nutrient concentrations among states. Points of different shapes and colors represent different nutrients and waterbody types. Only the variables from the best model based on AICc are shown. Lines represent 95% confidence intervals of the estimates. Negative values indicate a parameter was associated with reductions in nutrient concentration over time

Some of the variation in nutrient concentration trends was described by the input variables we identified (Table 2, Fig. 4). For all forms of N (NO_3_^−^, NH_4_^+^, and TN) in streams, the total amount of animal feed was identified as a predictor; the total proportion of variation explained by input variables was low for the dissolved forms (< 10%) and moderate for TN (24%) (Table 2). In lakes, the identified input variables described more variation in total nutrient concentrations (16–47%, Table 2). Agricultural variables (e.g., animal feed, fertilizer, and agricultural land use) were particularly important predictors of total nutrient concentration trends in both waterbody types. Change in human population and urban land use were only retained in the model for lake TN.

**Table 2:** Difference in Akaike Information Criteria adjusted for small sample sizes (ΔAICc) for policy models compared to the null model, which only included the nutrient input variables as predictors. A negative ΔAICc would indicate better model performance when a policy variable is included, and none of the policy variables improved the model fit

Nutrient	Terms in best model	Median *R*^*2*^	ΔAICc TMDL	ΔAICc 319	ΔAICc Nutrient criteria
Stream nitrate	Animal feed	0.08	2.27	2.27	2.24
Stream ammonium	Animal feed + Δ urban	0.03	2.27	1.94	1.20
Stream TP	Δ Agricultural land	0.13	1.79	1.98	1.79
Stream TN	Animal feed + Δ Animal feed	0.24	2.41	2.15	2.21
Lake TN	Agricultural land + Δ Animal feed + Δ Fertilizer + Δ Population + Δ Urban land	0.16	2.81	2.73	1.86
Lake TP	Δ Fertilizer + undeveloped land	0.47	2.39	2.14	0.93

**Fig. 5:**
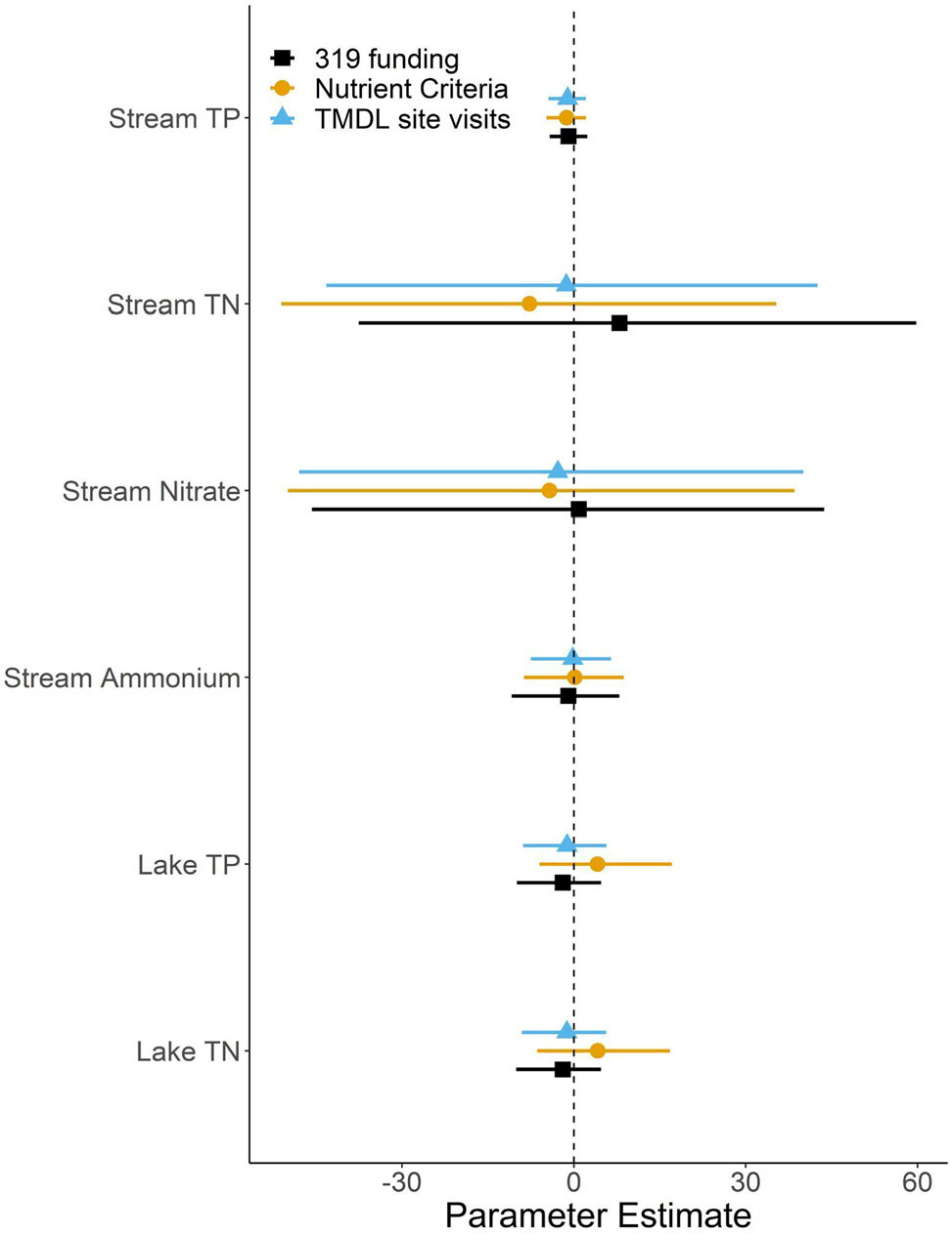
Parameter estimates of the effect of policy variables on trends in nutrient concentrations. Policy variables include the total state and federal spending on the 319 program normalized to the area of the state, the status of nutrient criteria development, and the total number of sites visited to assess status for TMDL relative to the area of the state. Values that are less than zero indicate that greater values of a policy variable (e.g., greater area-adjusted 319 spending) were correlated with greater reductions in nutrient concentrations at the state level

We did not find support for any of the policy variables as predictors of trends in nutrient concentrations (Fig. 5, Table 3). None of the policy variables reduced the ΔAICc of the model by more than two compared to the null model which only included the nutrient input variables (Table 3). Additionally, the 95% confidence intervals of the parameter estimate overlapped zero for each policy variable-waterbody-nutrient combination (Fig. 5, Table 3). Furthermore, the effect size estimates of the policy variables were well constrained. The lower bound of the confidence interval (2.5% quantile) represents the most generous effect that our models indicate these policies may have, and even these effects are relatively small. For instance, based on the lower end of our confidence intervals, our models indicate that adding one additional dollar of 319b spending per square kilometer of jurisdictional area ($343,350 in the average state) would reduce P concentrations by between 0.002 and 0.004 μg L^−1^ per year depending on the waterbody type, and would reduce N concentrations by between 0.004–0.018 μg L^−1^ per year depending on the waterbody and N form (Table 3). As an example, surveying one additional site per 10 km^2^ would move Florida from the state currently doing the least TMDL surveying per km^2^ to the top third of states (Fig. 1). According to the most optimistic parameter estimate for the effects of TMDL surveying, this substantial increase in surveying effort would lead to declines in P concentrations between 2 and 3 μg L^−1^ depending on the waterbody type and declines in N concentration between 3 and 19 μg L^−1^ per year depending on the form of N and waterbody type (Table 3). Finally, the effect of adding partial nutrient criteria for a single year could reduce P concentrations by between 1–2 μg L^−1^ per year depending on waterbody type and N concentration by between 3 and 14 μg L^−1^ per year depending on the form of N and waterbody type (Table 3). Though, we caution that these effects are the most optimistic, and confidence interval for each policy effect overlapped zero. Finally, we found that the effects of each policy overlapped zero when we evaluated models without nutrient input variables (Supplement S1), and models with trends in median, rather than mean, nutrient concentrations (Supplement S2).

**Table 3:** Quantiles of bootstrapped slope estimates from regressions relating change in nutrient concentration in states over time to policy variables, while accounting for changes in nutrient input variables. Policy variables were fit in their original units. The regression slope coefficient for 319 expenditure is expressed as the rate of change in nutrient concentration per dollar spent on a km^2^ of state jurisdiction area (μg L^−1^ year^−1^/$ km^2^). The nutrient criteria slope is expressed as the rate of change in nutrient concentration per 1 point nutrient criteria score (μg L^−1^ year^−1^/pts), which awards 2 points for every year a state has complete criteria for a nutrient in a waterbody type, 1 point for partial criteria, and 0 points for no criteria. The TMDL site visit slope is expressed as the rate of change in nutrient concentration per site visit in a km^2^ of state jurisdiction area (μg L^−1^ year^−1^/site visit km^2^). All parameter estimates overlapped 0

Policy	Nutrient type	Effect size		
		Lower CI (2.5%)	Median	Upper CI (97.5%)
319 spending	Lake TN	− 0.01	0.001	0.013
319 spending	Lake TP	− 0.004	− 0.001	0.002
319 spending	Stream ammonium	− 0.004	0	0.003
319 spending	Stream nitrate	− 0.018	0.001	0.018
319 spending	Stream TN	− 0.015	0.003	0.024
319 spending	Stream TP	− 0.002	0	0.001
Nutrient criteria	Lake TN	− 10.041	1.378	13.338
Nutrient criteria	Lake TP	− 1.759	1.047	4.85
Nutrient criteria	Stream ammonium	− 2.52	0.061	2.53
Nutrient criteria	Stream nitrate	− 14.389	− 1.164	11.356
Nutrient criteria	Stream TN	− 14.442	− 2.22	9.487
Nutrient criteria	Stream TP	− 1.368	− 0.366	0.654
TMDL visits	Lake TN	− 105.913	10.237	123.971
TMDL visits	Lake TP	− 34.992	− 3.656	21.786
TMDL visits	Stream ammonium	− 28.372	− 0.468	25.392
TMDL visits	Stream nitrate	− 188.042	− 6.665	161.087
TMDL visits	Stream TN	− 165.221	− 5.432	165.842
TMDL visits	Stream TP	− 16.652	− 4.099	7.928

## Discussion

We leveraged heterogeneity in state implementation of U.S. federal nonpoint source pollution provisions in the CWA to evaluate their efficacy in reducing nutrient pollution. We found no evidence in our analyses to support an effect of select CWA nonpoint source pollution provisions (Sects. 319 and 303) in reducing nutrient pollution in inland waters over a decade at the state scale; adding policy indicators (319b spending, numeric nutrient criteria development, or 303d site evaluations) to models describing state-level nutrient trends as a function of nutrient input variables (e.g., agricultural land use extent) failed to improve model fit. Furthermore, the most optimistic estimates of the effects of these policy variables on nutrient trends were very small relative to the scale of the problem of nutrient pollution. For example, adding an additional dollar of 319b grant spending per km^2^ would lead to reductions in stream TP of at most 0.02 μg L^−1^ per year. For context, a recent analysis found that the median concentration of TP in streams of the U.S. was roughly 91 μg L^−1^ (Manning et al. 2020), and thresholds for adverse biological effects of TP on stream organisms have been found at concentrations around 60 μg L^−1^ (Evans-White et al. 2013). We consider several nonconflicting possibilities for why we could not detect an effect of current federal policy: nutrient lags and legacies make progress undetectable, the best available data are insufficient to detect the effects of federal policy intervention, current implementation practices reduce the efficacy of the policies, and nonpoint source pollution control mechanisms in the CWA may contain structural issues that render them insufficient to reduce nutrient pollution.

Decades of fertilizer application in excess of crop demands has built up stores of nutrients in soil and groundwater that can be mobilized to waterbodies over a year after their application, introducing time lags in nutrient reductions following policy interventions (Sharpley et al. 2013; Van Meter et al. 2018). A model of a 500 km^2^ watershed in Iowa indicated that, following a total cessation of fertilizer application, a 50% reduction in streamwater nitrate could be achieved in 1 year in the scenario without N legacies but would take 19 years with N legacies (Ilampooranan et al. 2019). Legacy nutrient inputs may be contributing to minimal water quality improvements in the Chesapeake Bay (Chang et al. 2021; Stephenson et al. 2021), Mississippi River Basin (Van Meter et al. 2017), and catchments draining into the Baltic Sea (Chen et al. 2021), despite significant efforts to reduce nutrient inputs to these waterbodies. If nutrient concentrations are controlled by legacy inputs across the US, resulting lags in water quality improvement will mask or dampen positive effects of nonpoint source pollution provisions in the CWA.

The National Aquatic Resource Survey uses a randomized sampling design that allows unbiased estimates of changes in water quality over time in the Nation’s waters. However, not all aspects of nutrient input and policy implementation across US waterbodies are captured by the data used in our analysis, possibly limiting our ability to detect the effects of federal policy on nutrient trends. For example, factors like aging infrastructure could lead to increased nutrient inputs over time even without changes in population (Capps et al. 2020), and we were not able to account for factors such as these in our analyses. Also, quantifying the effects of policy on nutrient concentrations across states requires comparison of uniform data, but variation in data collection and reporting among states makes comparisons difficult. For example, the Performance Partnership Grant (PPGs) program allows states to combine 319b allocations with grants from other environmental programs to address environmental priorities identified by the state and agreed to by the EPA. PPGs aimed at reducing nonpoint source pollution may not be reflected in the Grants Reporting and Tracking System database from which we calculated 319b expenditures (U.S. EPA 2022a, M. Klasic 2022, *personal communication*), and thus, their effects are not accounted for in our analyses. Additionally, we were not able to consider policy developed and implemented on sovereign tribal lands, which could provide important insights and improve estimates of policy effects. Despite these challenges, some policy effects are well constrained by our models, and our models with only variables describing sources of nutrient input capture a modest amount of variation in nutrient trends. Finally, we contend that the state is the appropriate unit of analysis for CWA policy provisions as the CWA delegates authority to the states and the goal of the CWA is to protect and promote the integrity of the *Nation’s* waters (33 U.S.C. § 1313). Analysis of CWA nonpoint source programs implemented at the watershed scale may have larger effects than we estimated in our analysis. For example, several 319 projects have resulted in measurable declines in nutrient concentrations in individual waterbodies (U.S. EPA 2022d), but our results suggest that the current policy efforts that we examined in this study are having little effect at the state level. We note that we did not evaluate nonpoint source pollution policy efforts outside of those specified in the CWA, for example the U.S. Department of Agriculture’s National Water Quality Initiative which promotes voluntary nutrient pollution reduction efforts on agricultural lands. As additional and higher-resolution data become available, revisiting our analyses and evaluating additional programs may provide new insights.

Barriers to policy implementation may explain why there is no significant estimated effect of nonpoint source CWA programs on the reduction of nutrient concentrations. The U.S. EPA delegates authority to states to implement the policies we examined in this study. Yet, the success of state action to implement CWA policies and programs may be hampered without sufficient financial resources, necessary infrastructure, staff training, and accountability (Baehler and Biddle 2018; Bell et al. 2022). Furthermore, the economic recession that began near the beginning of our study period (2007–2008) led to reductions in staffing of some state and local agencies (Willard et al. 2012) and may have exacerbated issues of resourcing and implementation. Thus, while the effect of inadequate policy and challenges with implementing policy may be similar, the distinction between the two is critical as the remedies for these issues are distinct.

The final explanation we consider for our inability to detect the effects of CWA nonpoint source provisions on nutrient pollution are structural issues with the provisions themselves that may render them ineffective. We consider first that states have broad discretion over how they implement CWA nonpoint source pollution mandates. For example, states have flexibility in how they administer grants through the 319b program, with states like California implementing prescriptive regulation and states like Oklahoma relying on voluntary measures and cost sharing (Rotman et al. 2021). These different approaches to nonpoint source pollution management are unlikely to be equally effective in reducing nutrient pollution. While the EPA does have oversight authority for some aspects of state 303 and 319 implementation, this authority has been unevenly applied and hampered by legal challenges. A 2012 Government Accountability Office report found wide variation in EPA regional office oversight of state nonpoint source management programs and 319 grant project selection criteria (U.S. GAO 2012). Only some of the regional offices chose to be actively involved in developing criteria for project selection. Each regional office, however, almost always determined that the states under their jurisdiction had made satisfactory progress on the goals of their nonpoint source management programs, so as to not withhold 319 funds from underperforming states (U.S. GAO 2012). The EPA has been similarly sparing in exercising their oversight authority for water quality standards. Under Sect. 303, the EPA has the authority to prescribe water quality standards if they deem those promulgated by the state to be insufficient to meet the requirements of the CWA (U.S. EPA 2022b). This authority was partially exercised in the state of Florida; however, in this case, the EPA delayed the requirement of federal standards until Florida submitted revised numeric standards, three years after the agency made the determination that Florida’s original standards were insufficient. This process generated numerous legal challenges from both environmental advocates and industry groups (Copeland 2014). In the denial of a separate 2008 petition for rulemaking exhorting the EPA to promulgate numeric nutrient criteria for states that did not have them, the EPA asserted that they did not view promulgating criteria for states as the most effective tool for combatting nutrient pollution, in part because of the extraordinary resources required for such action (Weiss 2012).

A second structural issue is that states are not mandated to implement the nonpoint source pollution measures they are required to identify. While the CWA, and subsequent case law (e.g., Pronsolino v. Nastri 2002), compels states to develop TMDLs for impaired waters, states are not compelled by the law to implement the measures specified in these plans (Rotman et al. 2021). Currently, the only mechanism that the EPA has to compel states to address nonpoint source pollution is the “carrot” of 319 grant funds. As George Mitchell, sponsor of the 1987 amendment which added Sect. 319 to the CWA, declared, “If a state decides that it does not want a program to control nonpoint pollution, that is it” (133 Cong. Rec. S1968, 1987). Because of the lack of clear implementation accountability, these programs may not work to reduce nutrient pollution from nonpoint sources. In contrast, the CWA delegates much more authority to the EPA to regulate point source pollution, and these measures have been more effective in reducing point source discharges of pollution into waterbodies. For instance, spending on wastewater treatment plants under the CWA has reduced the biological oxygen demand and the dissolved oxygen deficit in waters downstream of wastewater treatment plant discharges (Keiser and Shapiro 2018).

We were unable to detect anticipated effects of nonpoint source CWA policy implementation factors on trends in nutrient concentrations in this study. Furthermore, we found that many states had increasing mean nutrient concentrations over the 10-year period we examined. Together, these findings indicate that the current scale and scope of nonpoint source pollution policy, implementation, and/ or management paradigm do not sufficiently reduce nutrient pollution in U.S. inland waters to meet the water quality goals outlined in the CWA (Rotman et al. 2021). The status quo of nutrient pollution is untenable; nutrient pollution is currently the leading cause of water quality impairment in the U.S., leading to reduced recreational opportunities, drinking water delivery disruption, loss of aquatic biodiversity, and increased water treatment needs (Conley et al. 2009; Rosemond et al. 2015; Dodds and Smith 2016; U.S. EPA 2016). However, there are exciting opportunities for creative action to improve water quality. For instance, there are technological advancements that can reduce the amount of nutrients applied (Hedley 2015), remove excess nutrients from the landscape (Speir et al. 2020), and recycle nutrients from human and livestock waste (Mihelcic et al. 2011; Harder et al. 2021). There are also opportunities for international diffusion of policy innovation. For example the European Union’s Water Framework Directive (WFD) includes catchment-based management, policy learning and adjustments, and integration of water policy into other policy domains, including agricultural policy and urban planning (Voulvoulis et al. 2017; Carvalho et al. 2019). The WFD specifically espouses an experimentalist approach to improved water quality, via provisional goal setting and revision that includes adjustments to policy (Voulvoulis et al. 2017). Such policy adaptation and revision are critical as baseline conditions continue to shift due to climate change (Lynch et al. 2022). Progress from this adaptive approach should be hastened by analysis and sharing of information and best practices among regions and state agencies. Without connections among environmental outcomes, policy, and management, ‘business as usual’ practices will likely lead to continued decline in water quality.

## Supplementary Information

Below is the link to the electronic supplementary material.

Supplementary file1 (PDF 598 kb)
