# Supplementary material for: Nonpoint source pollution measures in the Clean Water Act have no detectable impact on decadal trends in nutrient concentrations in U.S. inland waters
Source: Ambio. 2023 Jun 23;52(9):1475–87. doi: 10.1007/s13280-023-01869-6 (PMC10406756; doi:10.1007/s13280-023-01869-6)
Supplement: Supplementary file 1 — Supplementary file1 (PDF 598 kb) [file 13280_2023_1869_MOESM1_ESM.pdf]

Ambio

Electronic Supplementary Material

This supplementary material has not been peer reviewed.

Title: **Nonpoint source pollution measures in the Clean Water Act have no detectable impact on decadal trends in nutrient concentrations in U.S. inland waters**

Authors: Nathan Tomczyk, Laura Naslund, Carolyn Cummins, Emily Bell, Phillip Bumpers,

Amy Rosemond

Supplement S1:

Background:

The goal of the analysis in this manuscript was to evaluate the effect of federal water quality policy on mean nutrient concentrations in streams, rivers, and lakes, while accounting for factors which may alter nutrient inputs among states. However, if there are correlations among nutrient input and policy variables, from spurious or causal relationships, then including nutrient input variables in the same analysis as policy variables may reduce our ability to detect an effect of policy. Here we evaluated correlations among variables and refit our models to determine the effect of policy on nutrient concentration trends without accounting for nutrient input variables.

Methods:

We calculated Pearson's correlations among the policy variables and the nutrient input variables that were used in the models. We then reran the same analysis as described in this manuscript to evaluate the effect of policy trends in mean nutrient concentrations across states, without accounting for the nutrient input variables. We estimated effect sizes for each policy and nutrient-waterbody combination, and evaluated how the median effect sizes changed as a result of excluding nutrient input variables from the analysis.

Findings:

Of the combinations of nutrient input and policy variables that appeared in the final models, most had only weak correlation with Pearson's $r<0.35$ (Table S1). The exceptions were the correlation between the change in population and lake N criteria, and the change in urban land use and 319 spending which had correlations of 0.5 and 0.6 respectively (Table S1). Thus, because both of these correlations are positive, it is possible that increasing urban land use or

population masked effects of these policies on these nutrients. However, when we evaluated the effect of the policy variables on nutrient concentration trends using the models excluding nutrient input variables as predictors, we found that each effect sizes still overlapped zero. In half of the models, excluding nutrient input variables resulted in a higher median effect size, and half resulted in lower median effect size. While accounting for nutrient input variables does have a modest impact on the effect size estimates for these policy variables, the overall inference from this analysis—that the effects of these policies are not detectable at the state-level—is unchanged by excluding the policy variables from the analysis.

Table S1: Correlations among nutrient input and policy variables evaluated in this study. Numbers represent Pearson's correlations. Values in red represent combinations of variables that appeared in the final models.

	TMDL	319 Spending	Lake P criteria	Lake N criteria	Stream N criteria	Stream P criteria
Urban land use	0.41	0.77	0.36	0.05	0.01	0.12
Urban land use Δ	0.23	0.60	0.20	0.20	0.06	0.07
Agricultural land use	-0.28	-0.15	-0.18	-0.12	-0.23	-0.18
Agricultural land use Δ	-0.13	-0.25	-0.13	-0.09	0.20	0.05
Undeveloped land use	-0.04	-0.18	0.09	0.33	0.56	0.28
Undeveloped land use Δ	0.08	0.07	0.08	0.00	-0.25	-0.07
Fertilizer use	-0.20	0.01	-0.05	0.08	0.00	-0.07
Fertilizer use Δ	-0.25	-0.13	-0.03	0.15	0.09	0.01
Animal feed	-0.08	0.20	-0.18	-0.02	-0.09	-0.13
Animal feed Δ	-0.12	-0.31	0.01	-0.01	-0.01	0.07
Population	0.34	0.72	0.38	0.06	0.07	0.22
Population Δ	-0.03	0.19	0.21	0.50	0.38	0.13

Table S2: Quantiles of bootstrapped slope estimates from regressions relating change in nutrient concentration in states over time to policy variables, without accounting for changes in nutrient input variables. Policy variables were fit in their original units. The regression slope coefficient for 319 expenditure is expressed as the rate of change in nutrient concentration per dollar spent on a km^2 of state jurisdiction area (μg L^{-1} $year^{-1}$/ \$ km^2). The nutrient criteria slope is expressed as the rate of change in nutrient concentration per 1 point nutrient criteria score (μg L^{-1} $year^{-1}$/ pts), which awards 2 points for every year a state has complete criteria for a nutrient in a waterbody type, 1 point for partial criteria, and 0 points for no criteria. The TMDL site visit slope is expressed as the rate of change in nutrient concentration per site visit in a km^2 of state jurisdiction area (μg L^{-1} $year^{-1}$/ site visit km^2). Negative values are depicted in red for visualization purposes. All parameter estimates overlapped 0. Differences in the median effect size were calculated by subtracting the median effect size from this analysis from the analysis that included effects of nutrient input (Table 3).

Policy	Nutrient type	Effect size: Lower CI (2.5%)	Effect size: Median	Effect size: Upper CI (97.5%)	Difference in median
319 Spending	Lake TN	-0.011	0.000	0.012	0.001
319 Spending	Lake TP	-0.004	0.000	0.003	-0.001
319 Spending	Stream Ammonium	-0.002	0.000	0.003	0.000
319 Spending	Stream Nitrate	-0.016	0.002	0.019	-0.001
319 Spending	Stream TN	-0.021	0.000	0.019	0.003
319 Spending	Stream TP	-0.002	-0.001	0.001	0.001
Nutrient Criteria	Lake TN	-7.650	2.645	13.592	-1.267
Nutrient Criteria	Lake TP	-2.311	1.806	7.339	-0.759
Nutrient Criteria	Stream Ammonium	-2.219	0.271	2.730	-0.210
Nutrient Criteria	Stream Nitrate	-14.484	-1.614	10.604	0.450
Nutrient Criteria	Stream TN	-18.642	-3.178	9.986	0.958
Nutrient Criteria	Stream TP	-1.197	-0.123	0.895	-0.243
TMDL visits	Lake TN	-106.707	6.875	123.506	3.362
TMDL visits	Lake TP	-44.187	-2.927	30.138	-0.729
TMDL visits	Stream Ammonium	-20.332	4.480	28.460	-4.948

TMDL visits	Stream Nitrate	-191.817	-13.594	151.329	6.929
TMDL visits	Stream TN	-254.821	-36.413	137.331	30.981
TMDL visits	Stream TP	-19.079	-5.982	6.603	1.883

Supplement S2

Background:

Because distributions of nutrient concentrations can be skewed, evaluating the effect of policy on trends in median nutrient concentrations may provide distinct insight from evaluating the effect of policy on trends in mean nutrient concentrations. Thus, we calculated trends in median nutrient concentrations and then repeated our analyses to determine the effects of policy on those trends.

Methods:

We quantified trends in median concentrations using the *cont_analysis* function from the *sp_survey* package to calculate median nutrient concentrations for each state and each year. This function allowed us to account for the spatial and design weights essential to the National Aquatic Resources Surveys, which we also accounted for in our calculation of mean trends in our main analysis. We then used simple linear models to estimate trends in median nutrient concentrations for each nutrient and waterbody type in each state. We evaluated the effect of each policy on these trends as we did in the main text. Additionally, we compared the trends in median concentrations to the trends in mean concentrations using simple linear models.

Findings:

The trends in median nutrient concentrations varied among states, nutrients, and waterbody types (Figure S1, S2). The trends in median nutrient concentrations were generally similar to the trends in mean concentrations, with R^2 values greater than 0.4 for the relationship between trends in means and medians (Figure S3, Table S3). The exception was NH_4 in streams, which only had an R^2 of 0.24. However, while the trends in median nutrient concentrations were somewhat different than the trends in mean nutrient concentrations, the main conclusion about

the effect of each policy were similar with both methods. Specifically, the effect of each policy overlapped zero, indicating that we could not detect an effect of the policy in this analysis.

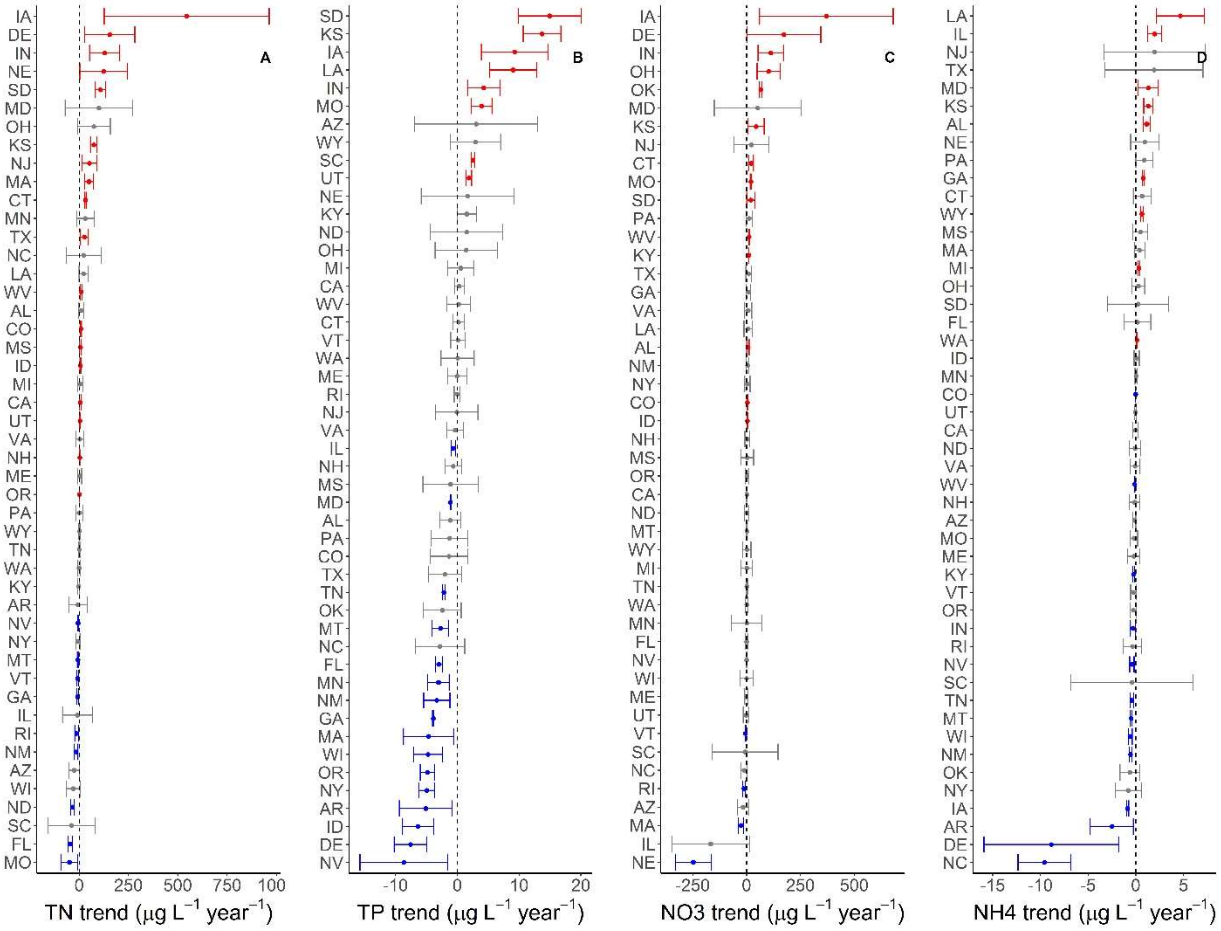

Figure S1: Trend in median concentrations of total nitrogen (TN, A), total phosphorus (TP, B), nitrate (NO3, C), and ammonium (NH4, D) in streams across states which were sampled 2008-2009, 2013-2014, and 2018-2019. In each panel, the states are sorted by their median nutrient trend. Points are presented with standard errors and colored based on whether the standard errors overlap zero. Blue points represent significantly declining median nutrient concentrations, red points represent significantly increasing nutrient concentrations and gray points do not have detectable changes in median nutrient concentrations.

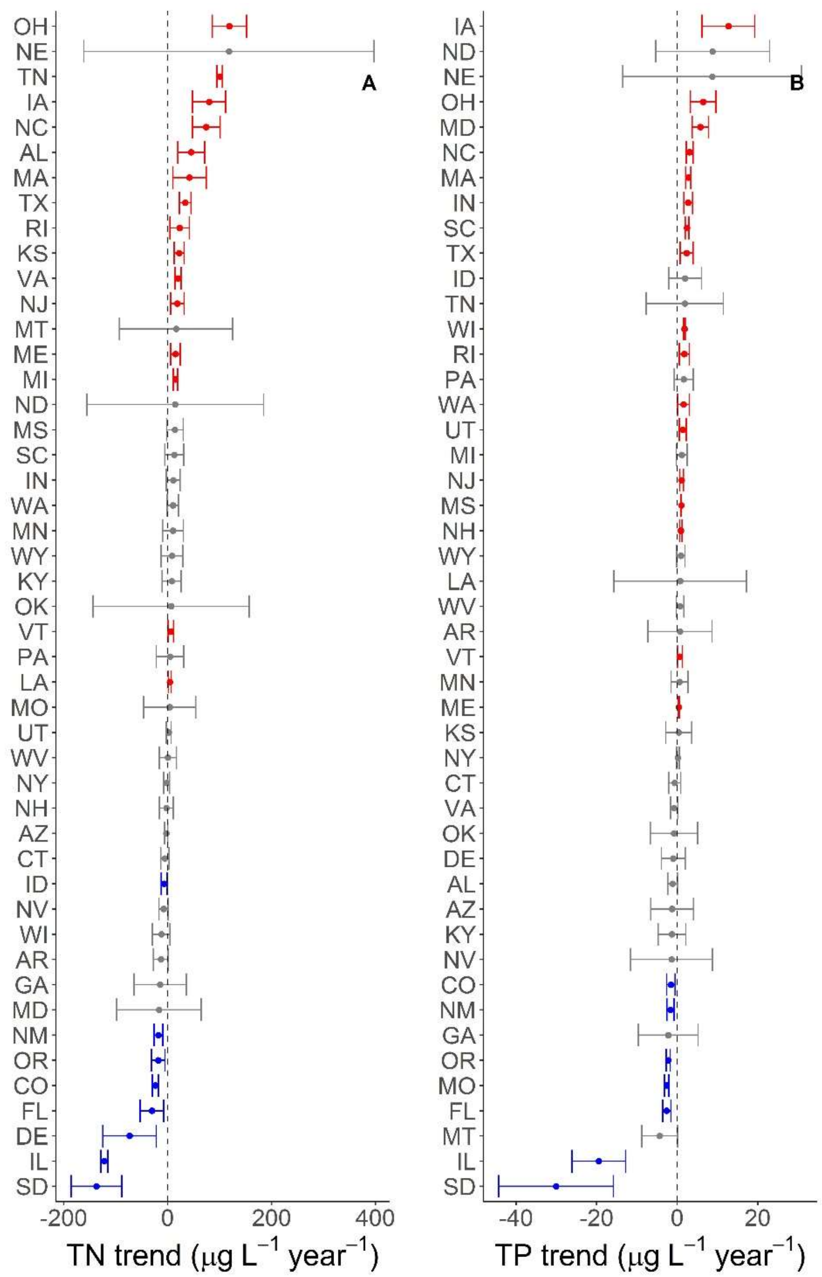

Figure S2: Trends in median concentrations of total nitrogen (TN, A) and total phosphorus (TP, B) in lakes and reservoirs which were sampled 2007, 2012 and 2017. In each panel, the states are sorted based on their nutrient trend. Points are presented with standard errors and colored based on whether they overlap zero. Blue points represent significantly declining median nutrient concentrations, red points represent significantly increasing nutrient concentrations gray points do not have detectable changes in median nutrient concentrations.

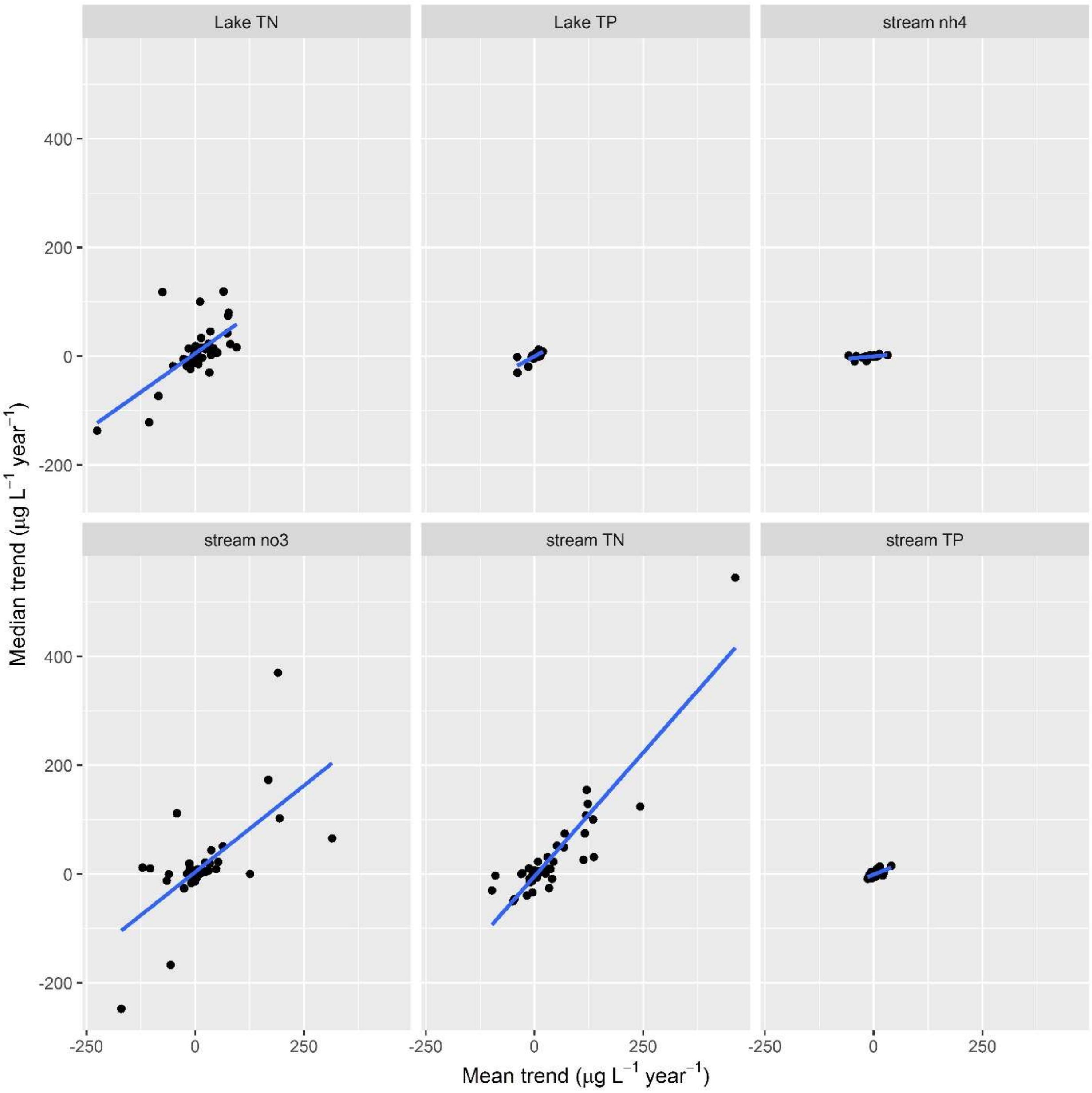

Figure S3: Comparison of trends in mean nutrient concentrations and trends in median nutrient concentrations. Each point represents the trend in one state, and the blue lines represent best fits. Equations for best fit lines are given in Table S3.

Table S3: Equations for lines of best fit associated with Figure S2. For each waterbody type and nutrient combination, the intercept and slope of the line of best fit, their associated standard errors, and the R^2 of the model is given.

Waterbody Type	Nutrient	Intercept (se)	Slope (se)	R^2
Lake	TN	5.30 (5.19)	0.57 (0.10)	0.42
Lake	TP	-0.05 (0.64)	0.44 (0.06)	0.52
Stream	NH_4	0.07 (0.28)	0.08 (0.02)	0.24
Stream	NO_3	3.97 (9.01)	0.64 (0.11)	0.41
Stream	TN	-3.95 (5.94)	0.91 (0.06)	0.83
Stream	TP	-0.75 (0.37)	0.32 (0.04)	0.42

Table S4: Quantiles of bootstrapped slope estimates from regressions relating change in median nutrient concentration in states over time to policy variables, while accounting for changes in nutrient input variables. Policy variables were fit in their original units. The regression slope coefficient for 319 expenditure is expressed as the rate of change in nutrient concentration per dollar spent on a km^2 of state jurisdiction area (µg L^{-1} $year^{-1}$/ $ km^2). The nutrient criteria slope is expressed as the rate of change in nutrient concentration per 1 point nutrient criteria score (µg L^{-1} $year^{-1}$/ pts), which awards 2 points for every year a state has complete criteria for a nutrient in a waterbody type, 1 point for partial criteria, and 0 points for no criteria. The TMDL site visit slope is expressed as the rate of change in nutrient concentration per site visit in a km^2 of state jurisdiction area (µg L^{-1} $year^{-1}$/ site visit km^2). Negative values are depicted in red for visualization purposes. All parameter estimates overlapped 0.

		Effect Size		
Policy	Nutrient Type	Lower CI (2.5%)	Median	Upper CI (97.5%)
319 Spending	Lake TN	-0.0093	0.0006	0.0103
319 Spending	Lake TP	-0.0012	0.0000	0.0011
319 Spending	Stream Ammonium	-0.0003	0.0000	0.0003
319 Spending	Stream TN	-0.0075	0.0034	0.0183
319 Spending	Stream TP	-0.0007	0.0000	0.0007
Nutrient Criteria	Lake TN	-6.5324	-0.5570	6.0061
Nutrient Criteria	Lake TP	-0.8644	-0.1594	0.5970
Nutrient Criteria	Stream Ammonium	-0.2436	-0.0347	0.1780
Nutrient Criteria	Stream TN	-11.1937	-2.5881	5.5362
Nutrient Criteria	Stream TP	-0.9501	-0.3046	0.3829
TMDL visits	Lake TN	-101.7651	-5.7059	82.1892
TMDL visits	Lake TP	-8.9661	1.2425	10.9323
TMDL visits	Stream Ammonium	-3.4046	-0.4067	2.5844
TMDL visits	Stream TN	-91.3376	12.8507	135.5380
TMDL visits	Stream TP	-7.5472	-0.5533	6.3495
